# Treating depression in clinical practice: new insights on the multidisciplinary use of trazodone

**DOI:** 10.3389/fpsyt.2023.1207621

**Published:** 2023-08-15

**Authors:** Umberto Albert, Carmine Tomasetti, Camillo Marra, Francesca Neviani, Alessandro Pirani, Daiana Taddeo, Orazio Zanetti, Giuseppe Maina

**Affiliations:** ^1^Department of Medicine, Surgery and Health Sciences, University of Trieste, Trieste, Italy; ^2^Azienda Sanitaria Integrata Giuliano-Isontina—ASUGI, UCO Clinica Psichiatrica, Trieste, Italy; ^3^ASL Teramo, Department of Mental Health of Teramo, Alzheimer Centre of Giulianova, Teramo, Italy; ^4^Neurology Unit, Fondazione Policlinico Universitario “A. Gemelli” IRCCS, Rome, Italy; ^5^Department of Neuroscience, Università Cattolica del Sacro Cuore, Rome, Italy; ^6^Center for Cognitive Disorders and Dementia, Chair of Geriatrics, University of Modena and Reggio Emilia, Modena, Italy; ^7^Center for Cognitive Disorders and Dementia, Health County of Ferrara, Ferrara, Italy; ^8^Alzheimer’s Association “Francesco Mazzuca”, Ferrara, Italy; ^9^Italian College of General Practitioners and Primary Care, Florence, Italy; ^10^IRCCS Istituto Centro San Giovanni di Dio Fatebenefratelli, Brescia, Italy; ^11^San Luigi Gonzaga Hospital, University of Turin, Turin, Italy; ^12^Department of Neurosciences “Rita Levi Montalcini”, University of Turin, Turin, Italy

**Keywords:** serotonin antagonist/reuptake inhibitors, comorbidity, psychiatric disorders, neurological disorders, dementia, primary care

## Abstract

Depression is estimated to be a leading contributor to the global mental health-related burden. The determinants of this huge prevalence lie in the fact that depressive symptoms may be comorbid in a wide variety of disorders, thus complicating and exacerbating their clinical framework. This makes the treatment of depressive symptoms difficult, since many pharmacological interactions should be considered by physicians planning therapy. Hence, depression still represents a challenge for both psychiatrists and other clinicians, in terms of its high rates of relapse and resistance despite well-established protocols. It is also complicated by the well-known latency in its complete response to current antidepressant treatments. In this context, the search for new strategies regarding antidepressant treatment is mandatory. Revising the use of “old” pharmacotherapies by considering their specific features may help to perfecting the treatment of depression, both in its standalone psychiatric manifestation and in the framework of other clinical conditions. Using a nominal group technique approach, the results of a consensus of expert physicians regarding the possible use of trazodone as a valuable strategy for addressing the “real world” unmet needs of depression treatment in different fields (psychiatry, primary care, neurology and geriatrics) is herein provided. This idea is based on the unique characteristics of this drug which delivers a more rapid antidepressant action as compared to other selective serotonin reuptake inhibitors. It also has pharmacodynamic malleability (i.e., the possibility of exerting different effects on depressive symptoms at different dosages) and pharmacokinetic tolerability (i.e., the possibility of being used as an add-on to other antidepressants with scarce interaction and achieving complimentary effects) when used in the milieu of other drugs in treating comorbid depressive symptoms. Moreover, the large number of formulations available permits finite dosage adjustments, and the use of trazodone for specific pathologies, such as dysphagia. Therefore, although additional studies exploring the real-world conditions of antidepressant treatment are warranted, experts agree on the idea that depressive disorder, in both its standalone and its comorbid manifestations, may surely take advantage of the particular characteristics of trazodone, thus attempting to reach the greatest effectiveness in different contexts.

## 1. Introduction

### 1.1. The challenge of real-world treatment of depression

Depressive disorders, together with anxiety disorders, are considered to be the leading contributors to the global mental health-related burden which, in turn, is the leading cause of global health-related problems, having an estimated 3.8% of adults affected worldwide, and a suicide rate of 700,000 people per year (1). In 2015, it was calculated that depressive disorders led to a global total of 5 million years lived with disability (YLD), thus being ranked as the major cause of non-fatal health loss (7.5% of all YLD) (2). Depression may show its specific set of symptoms (depressed mood, irritability, emptiness, loss of pleasure and interests, attention/concentration deficits, hopelessness, sleep disturbances, fatigue, pain, changes in appetite and weight) in the context of a classical major depressive disorder (MDD) or as a polar manifestation of bipolar disorder. It is, together with anxiety disorders, the most prevalent social disabling of psychiatric disorders. However, depressive symptoms may arise in the course of many clinical and neurological disorders, such as endocrine dysfunctions (e.g., hypothyroidism, cushing syndrome), cardiovascular disorders, stroke, Parkinson’s disease and major neurocognitive disorders (e.g., Alzheimer’s disease), thus exacerbating their progress and complicating their diagnosis and treatment as well as often impairing their outcome (3, 4). Consolidated evidence reports that an average of one in ten primary care patients present depressive symptoms (5). Moreover, since depression is significantly associated with chronic medical disorders, this frequent comorbidity, whenever misdiagnosed, may considerably impair the patient’s perception of medical care as well as increase the economic burden (6).

In addition to its prevalence and huge impact on clinical disability, depression also represents an important challenge for clinicians due to its low rates of response and remission, despite the increasing number of antidepressant treatment strategies. The largest pragmatic trial on real-world depression treatment, the so-called STAR*D (sequenced treatment alternatives to relieve depression) which analyzed the response to 4 levels of antidepressant treatment strategies in 2876 patients in both psychiatric and primary care centers, found that fewer than half of the patients enrolled responded to the first level of treatment. The remission rates were 28% in level 1, from 18% to 30% in level 2, and from 12% to 25% and from 7% to 14% in levels 3 and 4, respectively (7). Several studies re-analyzed the STAR*D data and findings, with the following main conclusions: (1) patients who did not achieve remission with the first level of treatment had scarce possibility of remission in the successive steps; (2) there were no significant differences in response rates amongst the different treatment strategies adopted for each level of treatment, and (3) the chances of a substantial treatment response significantly decreased after the first two levels of treatment (8).

Additional studies have demonstrated that 10% to 20% of patients may remain with depressive symptoms for a long time, even after a sequential treatment strategy (9); thus, one out of three patients fail to achieve full remission, possibly configuring so-called treatment resistance (10). Several RCTs (randomized clinical trials) regarding antidepressant treatments reported significantly higher rates of responses as compared to STAR*D (8). Therefore, it should be hypothesized that STAR*D failed to achieve ideal rates of antidepressant responses due to the “real-world” selection of patients who were enrolled in both primary and secondary care settings, as well as showing significant clinical comorbidities, thus giving a more realistic picture of the management of depression in clinical practice. Another explanation is that all patients, regardless of the clinical picture of depression (or subtype), received the same antidepressant as a first treatment while, in clinical practice, the choice of the right antidepressant for the right patient depends on several factors, including, for example, comorbid psychiatric and medical disorders (11).

### 1.2. The search for strategies in treating depression: new insights regarding trazodone

All the above-mentioned criticalities call for the use of new treatment strategies, both in depression as a standalone psychiatric disease and in the context of chronic medical illnesses in which it represents a synergic boost to the worsening of outcomes and to an increase in disability, mortality and economic burden (12).

Multiple strategies have been proposed to optimize the treatment of depression in order to both reduce the unresponsiveness rates and the impact of depressive symptom comorbidity on chronic illness disability. Although SSRI (selective serotonin reuptake inhibitor) antidepressants represent the first-line treatment for depression, they often fail to achieve acceptable remission, especially in comorbid depression (13). Thus, combination and augmentation strategies have been proposed, with mood stabilizers (e.g., lamotrigine), antipsychotics (e.g., quetiapine, aripiprazole), and GABA modulators (e.g., gabapentin) as well as using specific psychotherapeutic approaches (14). Comorbidity represents one of the most common cases of failure in depression treatment. In fact, the use of antidepressants in different contexts requires a more specific insight into the properties of any single drug.

Therefore, the revision of the use of “old” pharmacotherapies, which relies on the enhancement of their efficacy, based on new insights on their pharmacodynamic and pharmacokinetic properties, could represent another useful approach. According to this view, the present panel of experts has analyzed the possible use of trazodone as a valuable tool in approaching the unmet needs in the treatment of depression in different contexts, both as a standalone condition and as a comorbidity in medical, neurologic, and geriatric disorders.

Trazodone is a triazolopyridine derivative, developed in Italy by Angelini Research Laboratories in the 1960s as a second-generation antidepressant, based on the “mental pain” hypothesis of depression, and was the first non-tricyclic antidepressant approved in the U.S.A. in 1981 (15). Trazodone is the prototype of the serotonin antagonist/reuptake inhibitor antidepressants (SARIs), along with nefazodone, which have the pharmacological property of blocking 5HT2a/5HT2c serotonin receptors and inhibiting serotonin transporter (SERT) activity (16). To exert its complete action on SERT and 5HT2a/2c receptors, trazodone should be administered at medium/high doses. At low doses, instead, it preferentially antagonizes histamine 1 and alpha 1 adrenergic receptors, which are responsible—together with the potent blockade of 5HT2a receptors at these doses—for its anxiolytic and sedative/hypnotic effects (16).

The multimodality of trazodone use resides precisely in the possibility of exerting different effects on depressive symptoms at different doses, as well as on its malleability in an add-on with other SSRIs or SNRIs (serotonin/noradrenaline reuptake inhibitors) in order to achieve complementary effects. In fact, its anxiolytic/sedative/hypnotic properties may significantly increase remission rates in depression, by treating insomnia and anxiety in depressed patients, with higher efficacy as compared to SSRIs alone (17). In addition, the incremental recruitment of 5HT2a/2c receptors and SERT inhibition at higher dosages may incrementally exert antidepressant effects on mood and loss of energy by multiple—and not completely understood—mechanisms of action: the potentiation of serotonin’s inhibitory action via 5HT1a receptors (by means of a 5HT2a blockade) in specific neural circuits, with anxiolytic effects; the increase in 5HT1a-mediated gene expression which could lead to the production of neurotrophic factors associated with improvement in depression; the inhibition of glutamate release—via 5HT1a inhibitory action—in abnormally functioning cortical pyramidal neurons which are responsible for specific symptoms in depression (e.g., cognitive symptoms), and the disinhibition of noradrenaline and dopamine cortical release by means of 5HT2a and 5HT2c serotonin receptor blockade, having the final effect of relieving the main prefrontal-medicated symptoms of depression (16).

Currently, trazodone is marketed in three different formulations: immediate release (IR), prolonged release (PR), and once-a-day extended release (OAD). Trazodone IR has a rapid plasma peak (1 h) and a short half-life (6.6 h); trazodone PR has a slower plasma peak (4 h) and a longer half-life (12 h), and trazodone OAD shows a plateau plasma level for the entire day, with longer antidepressant concentration as compared to the other formulations (17). A considerable amount of data, starting from 1980s to nowadays, support the efficacy of trazodone in treating depression; it exerts comparable effects on all the other antidepressants when compared to a placebo, although with a scarcer antidepressant effect in head-to-head comparisons, due to the higher prevalence of side effects in older trazodone formulations with more drop-outs (18). The recent introduction of the OAD formulation helped to bypass these problems, granting a higher antidepressant efficacy than a placebo with a once-a-day administration, having side effects comparable to other antidepressants (19, 20). Moreover, trazodone displays high tolerability, even when administered to patients with comorbid clinical conditions, thus granting a safety profile in poly-pharmaco-treated depressed patients with chronic medical illnesses (21). Additional confirmation of trazodone tolerability has also been demonstrated in depressed patients suffering from COVID-19-related episodes (22). However, despite its safety and tolerability profile, trazodone, even in its OAD formulation, is currently under-prescribed in monotherapy, and often under-dosed (21), although its efficacy in real-world clinical practice when rightly administered has been repeatedly demonstrated, with rapid onset and prolonged decrease in depressive symptoms after 6 weeks, both in naïve patients and in patients unresponsive to previous treatments (23, 24).

Taken together, all these characteristics make trazodone a valuable and malleable choice in the treatment of depression since the pleiotropic and multimodal mechanisms of actions as well as the multiple formulations, may possibly result in the optimization of antidepressant treatment under specific conditions in which unmet needs must be satisfied.

### 1.3. Methodology and objectives

The following paragraphs are the result of a consensus of experts. Using the nominal group technique (NGT) (25), seven expert physicians in the fields of psychiatry, neurology, general practice, and geriatrics debated the issue of unmet needs in the treatment of depression in their different specific settings, and then on the possible use of trazodone, due to its specific characteristics, in those “real world” clinical practice contexts. In subsequent meetings, the experts were asked to review the literature regarding their specific field in order to generate ideas on the issue. The questions posed were: (1) What is the prevalence of depression/comorbid depression in the diseases you deal with in your clinical practice? (2) What are the unmet needs of depression in your clinical field? (3) What are the pharmacological issues which should be addressed in approaching these unmet needs? (4) Could trazodone, based on its particular characteristics, represent a useful tool for addressing unmet needs regarding depression in your clinical practice?

After having debated and ranked their ideas, the experts then decided to present the results of their consensus in the form of a narrative review, so as to facilitate accessibility and usability by the widest audience of physicians and clinical workers.

Therefore, in each of the following paragraphs, the objectives were to analyze the current unmet needs regarding the pharmacological treatment of depression in psychiatry, primary care, neurology and geriatric patients, according to the ideas of the expert physicians, supported by current scientific literature. Subsequently, the possible role of trazodone, due to its particular pharmacodynamic and pharmacokinetic properties in matching and filling those needs was discussed and supported by specific literature.

To the best of the authors’ knowledge, this is the first article which explores in detail the possible use of trazodone, traditionally considered an “old-fashioned” antidepressant drug, as a valuable tool in the treatment of depression in multidisciplinary contexts, due to its particular pharmacological features. However, the major limitation of this review relies in the fact that a significant number of studies for each discipline regarding this issue are still lacking, above all naturalistic studies exploring the real-word use of antidepressants in different contexts. We will try to debate on the specific limitations in each of the following paragraphs.

## 2. When antidepressants take too long: the unmet needs in the psychiatric treatment of major depressive disorder and the potential advantages of using trazodone

As previously discussed, depressive disorders affect 3.8% of adults worldwide, and represent the major cause of non-fatal health loss (1, 2). Moreover, an average of 1 out of 3 depressed patients fails to achieve correct remission using current antidepressant treatment strategies (10). The majority of the antidepressant drugs marketed have been developed based on the monoaminergic theory of depression which states that the pathophysiologic basis of depressive symptoms is a substantial depletion of monoamines (dopamine, noradrenaline, serotonin) in selected areas of the brain, a hypothesis which has long been supported by the antidepressant effects of the first drugs used [tricyclic antidepressants (TCAs) and monoamine oxidase inhibitors (MAOis)] (26). However, SSRIs, which represent the evolution of TCAs and make antidepressant efficacy “cleaner”—by reducing the onset of side effects—, do not seem to increase the response and remission rates in depression treatment as compared to older drugs. Moreover, a key limitation of antidepressant treatment is the substantial slowness of response; typically, the initial effects of any antidepressant drug begin to be observed in 2 weeks whereas it is possible that the full effects may not be reached before 6 to 8 weeks (27). A number of studies have pointed out that this delayed timing in antidepressant action might potentially lead to disastrous outcomes for patients, due to the sustained impairment which persists almost a month after the therapy has started (28). An increased risk of suicide in the first month of treatment, or even in the first week, has been reported (29, 30). Moreover, persistent impairment may lead to a loss of social and work functioning, with a progressive worsening of the quality of life (31). In turn, all these disabilities may impact on the same course of the disease, worsening and prolonging it, and on the economic burden related to depression.

Rapid relief of depressive symptoms, by contrast, has been associated with more favorable outcomes. Increasing evidence has suggested that SSRIs may show their first antidepressant effects by the end of the first week of treatment, with a decreasing rate of effects continuing until 6 weeks (32). Clinically, the early improvement of depressive symptoms (up to 2 weeks) has been associated with successful treatment, with a sustained response and with higher rates of remission (33).

An increasing number of studies have demonstrated that antidepressant action may be accelerated by means of different strategies. For example, physical treatment, such as electroconvulsive therapy (ECT), may also achieve rapid and sustained responses in depressed patients not responsive to pharmacological treatment (32). The recent development of non-monoaminergic antidepressant drugs, such as ketamine [an N-methyl-D-aspartate (NMDA) glutamate receptor antagonist], has been a milestone in fast-acting antidepressant treatment, paving the way for future strategies other than monoamine modulation (34). However, these new treatments will require additional development and studies in order to find their appropriate positioning in antidepressant treatment algorithms.

The new discoveries regarding the specific roles of serotonin receptor subtypes in the pathophysiological mechanisms of depression have paved the way for the development of new monoamine-based treatments with enhanced antidepressant efficacy, based on their multimodal modulation of these receptors (27). For example, the finding that the addition of pindolol—a 5HT1a serotonin receptor antagonist and beta-adrenoreceptor antagonist—may accelerate and increase the antidepressant effect of SSRIs has focused attention on 5HT1a presynaptic receptors as an essential target of antidepressant treatment (35). In light of these findings, older antidepressant drugs, such as trazodone, may be re-positioned as a valuable treatment strategy for depression based on its specific pharmacological properties which may help to fill the time gap of antidepressant action (36).

Recent studies have reported that trazodone, in addition to its well-known action on 5HT2a/2c serotonin receptors and on SERTs, may exert partial agonist action on 5HT1a serotonin receptors, and also show a medium-high affinity for 5HT7 serotonin receptors (37). The concurrent blockade of 5HT2a/2c receptors and of SERTs, and the modulation of 5HT1a and 5HT7 receptors may enhance and accelerate the antidepressant action of trazodone by increasing serotonin postsynaptic action and the subsequent disinhibition of dopamine and noradrenaline cortical release, together with the previously described glutamate-modulated neurotrophic factor gene expression (16, 38). Two studies have corroborated these preclinical data with the clinical observation of the fast-acting antidepressant action of trazodone. Sheehan et al. (19) demonstrated that trazodone OAD (150–225 mg/day) may induce a substantial reduction in depressive symptoms-as measured with the 17-item Hamilton depression rating scale (HDRS17), within a week of treatment, and that these effects may persist until the end of the study (56 weeks). In a more recent study, Fagiolini et al. (39) reported a faster antidepressant response (within 7 days) in patients treated with trazodone OAD (150 mg/die) as compared to venlafaxine XR (75 mg/die). At the end of the studies, the effects of the two antidepressants were similar. A recent HDRS17 factor re-analysis of the two studies found that the faster antidepressant effects of trazodone were not only exerted, as expected, on the sleep component of the depressive symptoms, but also on the cognitive aspects of depression (36). These results seemed to be in line with a previous report of the rapid antidepressant efficacy of trazodone OAD (1 week) in a clinical practice setting, as measured using the Montgomery–Åsberg depression rating scale (MADRS) (24). Moreover, early studies had already found that trazodone IR resulted in a greater reduction after 1 week of treatment using the HDRS as compared to bupropion and fluoxetine (40, 41) as well as to trazodone PR vs. a placebo (42).

Therefore, trazodone may represent a useful treatment for filling the gap in antidepressant timing of action, helping to already improve specific depressive symptoms in the first week of treatment. The multimodal modulation of serotonin receptors by trazodone may grant rapid relief of sleep disturbances and anxiety which represent highly disturbing symptoms in depression, the remission of which may, in turn, sustain and consolidate the long-term response to other psychopathological aspects of depression during the course of treatment. Moreover, the earlier impact of trazodone on the cognitive aspects of depression, most likely due to its modulation of 5HT7 receptors (43), not only synergistically improves its fast antidepressant action (44), but also lays the first stone in developing an antidepressant strategy which might reduce the impact of depression on the social and work functioning resulting from the progressive cognitive impairment of affected patients.

Recently, particular attention has been paid to pharmacogenetics, with regard to the tailoring of antidepressant prescriptions. Cytochrome polymorphisms have been demonstrated to scarcely influence trazodone pharmacokinetics (45), although lower bioavailability may be found in women, probably due to increased CYP3A4 activity (46). Moreover, a polymorphism in P-glycoprotein *ABCB1* (ATP binding cassette subfamily member B1, a pump exporting substance outside the cells, the T/T allele of C3435T polymorphism which has been associated with slow metabolizers) has been associated with higher rates of low blood pressure and prolonged QTc interval by trazodone (47). Thus, considering these variants may further help to improve efficacy and tolerability of trazodone usage in depressed patients.

## 3. A close look at the frontline: the unmet needs of depression in primary care patients and the possible use of trazodone

Depression represents one of the ten the most frequent illnesses affecting the patients general practitioners (GPs) see in their office (48–50). An estimated 27% of general outpatients show depressive symptoms (6), with high prevalence in patients diagnosed with cardiovascular diseases (11%–42% in heart failure, 28% in myocardial infarction), metabolic diseases (23%–34% in type 2 diabetes mellitus) and cancer (16.3%) (51). Even if the long relationship of caring for patients by GPs favors a narrative medicine approach to their patients (52), depression remains underdiagnosed (53) probably due to the heterogeneity of the population which is under their responsibility: young, adult and old outpatients (54) with different family and social conditions (single, divorced, widowed, retired/lonely, immigrant, insufficiently employed, quarreling/trouble with family) (55), work problems, sexual orientation and gender identity (56) as well as with several unmet needs and comorbidities, especially in elderly patients (54, 57, 58).

Once the GP has supposedly diagnosed depression, the target outcome should be to obtain functional remission (59, 60) and to achieve complete psychosocial recovery of the patient (61). According to the most recent scientific evidence, the cornerstone strategy for reaching clinical effectiveness is a personalized (“tailored”) treatment based on a decision tree (62) which should primarily include the patient in the choice of medication(s), addressing potential therapeutic effects, likely time to respond, general tolerability and possible adverse effects (e.g., weight gain, sexual adverse events, cardiovascular adverse events) (63).

Trazodone is often selected as a first choice antidepressant in primary care settings due to its multimodal action (anxiolytic, hypnotic, antidepressant) exerted at different dosages as well as to its wide range of available formulations, its rapid efficacy, a low rate of adverse events, and less interaction with other drugs, all of which make trazodone extremely useful and malleable in high comorbid patients (21, 36, 64).

Secondarily, but not less importantly, the correct management of titrating the medications by GPs, and the correct timing of assessment, may help to increase the possibility of long-term effective treatment, favoring treatment adherence (62) as well as reducing discontinuation rates (65).

Of the factors determining unsuccessful treatment, the antidepressant tachyphylaxis plays an important role (66, 67). Antidepressant tachyphylaxis has been described as the condition in which a patient may lose a previously effective antidepressant effect, even while staying on the same dosage of a drug for maintenance treatment (66). Serotonin receptor downregulation (68), alterations of neurogenesis (69) and of dendritic hippocampal arborization (70) by chronic antidepressant exposure are all pathophysiological mechanisms which are supposed to underlie antidepressant tachyphylaxis, which often force physicians to switch antidepressants, with possible withdrawal symptoms. Withdrawal may occur with all SSRIs, serotonin-noradrenaline reuptake inhibitors (SNRIs), benzodiazepines and antipsychotics, with symptoms resembling a relapse or recurrence of the original illness (71). Trazodone has been said to induce fewer withdrawal symptoms, due to its specific mechanisms of action (21), thus appearing to be a valuable choice of treatment for managing depression in primary care settings.

## 4. Taking care of an impaired brain: the unmet needs of depression in neurological disorders and the possible benefits of trazodone

Different papers have reported that the prevalence of depression related to neurological disease varies significantly. This is mainly due to the different methods and criteria used. A major problem related to the diagnosis of depression associated with neurological disorders is the overlap between symptoms usually due to the depression and those which are a consequence of the disease *per se* (72). Some somatic items, such as “psychomotor slowdown,” “fatigue,” “physical anxiety,” and “insomnia” have low discriminative properties but a high prevalence among neurologic disorders. Thus, the majority of those patients could result as being considered depressed at the MADRS or HDRS, although there is a lack of a real feeling of depression, usually with a non-pathological level of thoughts of suicide, feelings of guilt, poor self-esteem and loss of motivation and pleasure which are typical symptoms of “endogenous depression.” On the other hand, patients affected by depression could be misdiagnosed due to the fact that the majority of the symptoms are often considered primarily to be symptoms resulting from the course of neurological diseases (73).

The correct analyses of the symptomatologic presentation of depression among neurological disorders is of paramount relevance in order to prescribe the correct treatment to the patients which could be more oriented, in some cases, to treating either the physical or the emotional symptoms of depression alternatively.

Several neurological conditions are associated with depression. Three neurological diseases in which depression is often associated with the primary neurological disorder will additionally be considered. Apart from dementia, depression is a condition usually observed in Parkinson disease (PD) (74), multiple sclerosis (MS) (75) and post-stroke disease (PSD) (76).

Mood and behavioral disorders are part of the core symptoms across all stages of PD. Depression in PD is very common, and the prevalence rate of major depression in PD ranges from 4% to 70% with an average prevalence of 40% (72). This wide fluctuation could be due to the factors mentioned above, namely the overlap of some symptoms which are characteristic of both depressive disorders and PD, such as fatigue, loss of energy, loss of appetite, psychomotor slowing, amimia, bradyphrenia, difficulty in concentration, and insomnia. In a study on 169 patients with PD, Leentijens et al. (77) found that, when using the Hamilton depression rating scale (HAM-D scale) as a screening test, “suicide attempt” (with a low prevalence of 20 percent) was the item which best differentiated depressed from non-depressed PD patients, while somatic items, such as “psychomotor slowdown,” “fatigue,” “physical anxiety” and “insomnia,” although very prevalent, had low discriminative properties, being present in both PD with and without depression.

The majority of patients with PD meet the criteria of anhedonia more often than depressed mood in DSM-5. This is in agreement with the more severe impairment of the dopaminergic system which affects motivation/rewarding more than the serotoninergic system in these patients (78, 79).

Similar to what has been observed in Alzheimer’s disease, there is a direct relationship between depression and cognitive disorders (80). The cognitive domains mainly affected by depression in PD patients are the working memory and the executive functions; this cognitive impairment, as for major depression, is reasonably sustained by the involvement of the dopaminergic system (81).

According to this background, the treatment of PD associated with depression is critical for the quality of life of these patients; however, the choice of the antidepressant requires taking into account the neurotransmitter mainly involved in this disease.

The treatment of the patients is based on the replacement of dopaminergic transmission, antidepressant medications and psychotherapy. To date, there are no RCTs for dopamine agonists and tricyclic antidepressants (TCAs). Some RCTs for SSRIs and SNRIs have shown a satisfying efficacy for PD patients with depressive symptoms. The majority of them produce side effects on both the motor symptoms and the vegetative disorders associated with the disease; SSRIs could also increase apathetic disorders and emotional indifference among PD patients. Moreover, potential interaction with the other drugs used for PD should be considered. In the current guidelines, no statements, algorithms or recommendations are given for the diagnosis and treatment of depression in PD (73).

Although there are no specific studies regarding this issue, considering its lack of modulation of dopamine receptors and its activity on 5HT2a/c (82), very poor interference with motor symptoms in PD patients and good action against anxiety and insomnia, which are usually present in PD patients with depression, should be expected. Parkinson patients treated with trazodone show a clear benefit in motor symptoms with a reduction in dyskinesia and dystonia with respect to what has been reported for other antidepressant drugs used for PD in both animal (83) and human (84) models. Moreover, trazodone has a low level of side effects on the neurovegetative disorders (constipation and hypotension) usually characteristic of PD (21). Therefore, trazodone may represent a valuable choice in the treatment of depression in PD with a low level of side effects.

Multiple sclerosis is another neurological condition often associated with depression. Depression is more frequent among people with MS than it is in the general population, and almost 50% of MS patients experience an episode of major depression in the course of the disease (75). The inflammatory condition and the side effects of some medications (corticosteroids and interferon) may trigger or worsen depression in susceptible individuals (85, 86). However, many factors seem to contribute to developing depression. The difficulty of coping with the stigma of the disease is a stressful condition across all stages of the disease, at the beginning (following diagnosis), during an exacerbation of the disease or a hospital admission, or when a major change in functionality occurs. From another perspective, depression could be caused by the disease process itself. In particular, controlling the inflammatory condition and damage to the brain areas involved in emotional expression can result in a variety of behavioral and mood changes. A strong link between fatigue and depression in MS makes it difficult to tell the two conditions apart; this is also a source of confusion when applying depression scales to MS patients (87). Therapy for depression in MS deserves undivided attention since several studies have reported the protective effect of antidepressant therapy on MS progression and relapsing episodes due to stressful life events (88).

A recent and extensive review has proven the effect of antidepressants (SSRIs, SNRIs, TCAs) in reducing some inflammatory markers of the disease in both *in vivo* and *in vitro* mouse models (89).

In spite of the widespread use of antidepressant drugs, and the occurrence of depression in MS, there is a lack of specific RCTs regarding the issue. According to a recent review (90), depression treatment has been observed in 57.25% of MS patients. Fluoxetine is the most prescribed individual antidepressant (25.4%); trazodone is used in only 5.8% of cases, mainly in older patients and when patients are also affected by chronic diseases. Age and number of chronic diseases are significant predictors of depression in MS patients. This observation seems to highlight the idea of using trazodone in older MS patients owing to its safety and lack of interaction; this makes its use easier in patients undergoing polypharmacotherapy.

One of the most important neurological conditions found in MS patients is PSD; PSD is present in at least 30%–40% of survivors from stroke and has a deleterious effect not only on motivation, but also on the cognitive functions and rehabilitative outcome of these patients (91, 92). The main biological theory of PSD is the amine depletion hypothesis. Conceivably, ischemic lesions interrupt the projections ascending from the midbrain and the brainstem, leading to decreased bioavailability of the biogenic amines, such as serotonin (5HT), dopamine (DA) and norepinephrine (NE). Some authors have, for the most part, ascribed a multiple hypothesis to be the cause of depression, also involving the psychological adaptative response to the handicap. In addition to what has been described above, PSD represents a complex phenomenon, also including neurotrophin signalling, neuroinflammation, hippocampal neurogenesis, and endocrine activation of the hypothalamic-pituitary-adrenal (HPA) axis.

To date, SSRIs have especially been proven to be clinically active in preventing and treating PSD; however, no RCTs are available regarding these drugs; a risk/benefit effect (also considering bleeding and intracerebral hemorrhage) has not been explored. There is evidence supporting the fact that SSRIs exert a direct effect on serotonin but also a pleiotropic mechanism of action including anti-inflammatory and up-regulation of the neurotrophins important for neurogenesis and functional recovery (93).

Trazodone received less attention in the past 20 years; however, several clinical trials had already shown that trazodone improved depression and rehabilitative outcome in PSD patients with a low risk of side effects (94).

An important RCT by Reding et al. (95) had already used trazodone at a dosage of 200 mg/die. Patients with either a clinical diagnosis of depression or abnormal Zung depression scores showed a consistent trend toward greater improvement in the Barthel activities of daily living (ADL) scores with trazodone than with a placebo. The biological effect was proven by an abnormal dexamethasone suppression test associated with significant improvement in the Barthel ADL scores of patients receiving trazodone.

Although there are no data available regarding the different outcomes using trazodone or SSRIs in clinical practice, evidence has suggested that the use of trazodone was not inferior to other anti-depressants regarding either depressive symptoms or rehabilitative outcome; there was a minor risk of bleeding in patients with PSD using trazodone (96).

## 5. When depression gets old: unmet needs of depression in the elderly, and the possible use of trazodone

Depression and anxiety disorders in the elderly are common and under diagnosed. Elderly patients often present more somatic than psychiatric symptoms, thus making the right diagnosis difficult for the general practitioner (54). The World Health Organization (WHO) defines depression as one of the four “giants” of geriatrics. It is estimated that 10 out of 100 people over 65 years of age suffer from depressive symptoms and perceive their psychological well-being as compromised for an average of 18 days in the month preceding being interviewed (1). When approaching depression in elderly patients, physicians often find themselves dealing with a complex condition characterized by suffering, abandonment, and generally compromised functioning. Encouraging physicians to take care of the worldwide condition of depressed elderly people, Cassel (97) said: “suffering is experienced by persons, not merely by bodies, and has its source in challenges that threaten the intactness of the person as a complex social and psychological entity. Suffering can include physical pain but is by no means limited to it. The relief of suffering and the cure of disease must be seen as twin obligations of a medical profession that is truly dedicated to the care of the sick. Physicians’ failure to understand the nature of suffering can result in medical intervention that (though technically adequate) not only fails to relieve suffering but becomes a source of suffering itself.”

Late-life depression is often diagnosed in the context of multiple comorbidities, both medical and neurological (see the next section). In fact, cardiovascular (mainly heart failure) and cerebrovascular (e.g., atherosclerosis) diseases, thyroid dysfunction, adrenocortical disorders, diabetes, vitamin B12 and folic acid deficiency as well as malnutrition represent the most common medical conditions associated with geriatric depression (98). Also, multiple treatments, which are often used to manage these conditions in elderly people (e.g., antihypertensive medications) may also contribute to exacerbate, or to develop, depressive symptoms (99). Last but not least in importance, poor psychosocial conditions, such as low economic status, abandonment, isolation and relocation, and senile decrepitude (i.e., the loss of abilities and pleasure), which often stay with people in the late stages of their lives, may trigger depressive symptoms or worsen pre-existing depression (99).

In the elderly above all, the spectrum of depression takes on a wide range of manifestations. Taking into consideration the symptoms listed in the DSM-5 for major depressive episodes, there are 227 possible symptom profiles, within the same diagnosis (100).

In this context, trazodone, due to its multimodal action and its flexibility, represents a clear resource. Various studies have reported the considerable safety of trazodone which has scarce anticholinergic effects (e.g., constipation, urinary retention) (21) and a large tolerability, even when prescribed in poly-treated patients (101).

Insomnia and sleep disturbances may represent a particularly prevalent disorder in the elderly population, having a clear bidirectional relationship with late-life depression, being exacerbated in depressed patients and in turn triggering and worsening depressive symptoms when unmanaged (102, 103). Low dosages of trazodone (25 to 100 mg) may be helpful in secondary insomnia in elderly depressed patients, having a scarce impact on sleep architecture (103, 104), also when administered to patients with severe comorbid pathologies, such as advanced cancer (105). These data suggested that trazodone may represent a valuable choice for elderly patients having a complex medical history with multiple clinical comorbidities.

## 6. When depression gets old…and sick: the unmet needs of depression in geriatric disorders (dementia and delirium), and the possible use of trazodone

Dementia is a common condition in elderly people. In 2020, over 55 million people were diagnosed with dementia, and the WHO estimated that this number would almost double in 20 years, with high prevalence in developing countries (106). The relationship between dementia and depression has been widely studied; nevertheless, the nature of this association is still unclear (107). Depression has been reported in 30% of people diagnosed with vascular dementia or Alzheimer’s disease, and in 40% of patients diagnosed with dementia associated to Parkinson’s od Huntington’s diseases (108). Moreover, it is not infrequent that depression might be the consequence of a diagnosis of dementia, mainly related to the emotional impact of cognitive impairment and to the patient’s insight (109). In turn, the awareness of memory loss and cognitive impairment, the social stigma related to dementia, the lack of ability and the patient’s role in the society may cause depression (110).

On the other hand, early-life depression is often associated with a high risk of developing dementia (111). Evidence of vascular disease, hippocampal atrophy, alterations in glucocorticoid steroid levels, inflammatory changes and a deficit in nerve growth factors are common in both diseases (111).

Of the many manifestations of depression, one of the most interesting, and disturbing, is delirium which seems to have, in turn, a deleterious influence on the course of depression. People affected by depression have a high risk of delirium, and depression is a frequent sequela of delirium (112).

Depression and delirium are frequent conditions and have a very catastrophic impact on patient wellness, survival and daily living maintenance, especially when elderly patients with dementia are hospitalized (113). It is very important to treat these conditions immediately in order to reduce patient discomfort and improve hospitalization outcomes (114).

When the triad of depression, dementia and delirium occurs, their treatment should be based on a multicomponent approach characterized by pharmacological and psychosocial interventions (115, 116).

,Trazodone has demonstrated efficacy in reducing depressive symptoms, anxiety, insomnia, agitation and apathy in both dementia and delirium (117). The low anticholinergic effects also make trazodone safe in elderly people with cognitive impairment and delirium (118–121). Moreover, recent studies have demonstrated that patients with dementia taking trazodone may have delayed decline in cognition (122).

The many formulations of trazodone available can be used to treat different needs in patients with dementia. For example, droplets may be very useful for patients having dysphagia (123). In fact, OAD technology might be more indicated to treat depression in people with early stage dementia, thanks to its rapid onset of action, the once-a-day administration and the safety of the profile (124). Low doses of trazodone (50–100 mg) by i.v. or i.m. infusion may reduce agitation and have few side effects; they have been reported to be particularly useful in hospital setting (125).

## 7. Conclusion

Despite many years of research regarding the issue, depression still represents a huge challenge for clinicians, due to its pleiotropic manifestations, and the scarce treatment response as well as its low rate of complete remission and recovery, and the high rates of recurrences and chronicity. An important bias in the scientific literature regarding depression is the scarceness of “real world” clinical trials. Depression may appear in the context of multiple diseases, worsening and complicating their outcomes, and making its treatment even more difficult as compared to its standalone manifestation and major depressive disorders. In the search for new therapeutic strategies, it is worth glancing at “old” treatments, which could be repositioned according to their specific receptor profiles. Therefore, the authors herein reviewed the use of trazodone, its specific pharmacological characteristics and its many formulations which can be used to improve depression outcomes, above all in real world clinical practice, both in standalone psychiatric major depression and in other clinical diseases in which depressive symptoms often show up in the course of complicated illnesses requiring *per se* multitargeted pharmacotherapy. Major depressive disorder treatment can take advantage of the rapid onset of action of trazodone whereas neurological disorders, dementiae and diseases of the elderly may benefit from trazodone treatment when depression occurs. This is a result of its particular malleability and a lack of meaningful interactions as well as its large number of formulations which permit multiple dosage adjustments and its use under particular conditions (i.e., dysphagia associated with dementia). Clearly, additional studies which explore the real-world conditions of antidepressant effects are desirable, with the aim of achieving the utmost effectiveness in the treatment of depression in all contexts of its manifestation.

## Author contributions

UA and GM developed the original idea. CT wrote the first and the last draft of the manuscript. UA, CT, CM, FN, AP, DT, OZ, and GM equally contributed to the conception, contributed to the writing, and revisions of the paper. All authors contributed to the article and approved the submitted version.

## Funding

The authors declare that this study received funding from Angelini Pharma. The funder was not involved in the study design, collection, analysis, interpretation of data, the writing of this article, or the decision to submit it for publication.

## Conflict of interest

The authors declare that the research was conducted in the absence of any commercial or financial relationships that could be construed as a potential conflict of interest.

## Publisher’s note

All claims expressed in this article are solely those of the authors and do not necessarily represent those of their affiliated organizations, or those of the publisher, the editors and the reviewers. Any product that may be evaluated in this article, or claim that may be made by its manufacturer, is not guaranteed or endorsed by the publisher.
